# 4-Bromophenacyl Bromide Specifically Inhibits Rhoptry Secretion during *Toxoplasma* Invasion

**DOI:** 10.1371/journal.pone.0008143

**Published:** 2009-12-02

**Authors:** Sandeep Ravindran, Melissa B. Lodoen, Steven H. L. Verhelst, Matthew Bogyo, John C. Boothroyd

**Affiliations:** 1 Department of Microbiology and Immunology, Stanford University, Stanford, California, United States of America; 2 Center for Integrative Protein Science Munich, Chemistry of Biopolymers, Technical University Munich, Freising, Germany; 3 Department of Pathology and Microbiology and Immunology, Stanford University, Stanford, California, United States of America; Louisiana State University, United States of America

## Abstract

*Toxoplasma gondii* is a eukaryotic parasite of the phylum *Apicomplexa* that is able to infect a wide variety of host cells. During its active invasion process it secretes proteins from discrete secretory organelles: the micronemes, rhoptries and dense granules. Although a number of rhoptry proteins have been shown to be involved in important interactions with the host cell, very little is known about the mechanism of secretion of any *Toxoplasma* protein into the host cell. We used a chemical inhibitor of phospholipase A2s, 4-bromophenacyl bromide (4-BPB), to look at the role of such lipases in the secretion of *Toxoplasma* proteins. We found that 4-BPB was a potent inhibitor of rhoptry secretion in *Toxoplasma* invasion. This drug specifically blocked rhoptry secretion but not microneme secretion, thus effectively showing that the two processes can be de-coupled. It affected parasite motility and invasion, but not attachment or egress. Using propargyl- or azido-derivatives of the drug (so-called click chemistry derivatives) and a series of 4-BPB-resistant mutants, we found that the drug has a very large number of target proteins in the parasite that are involved in at least two key steps: invasion and intracellular growth. This potent compound, the modified “click-chemistry” forms of it, and the resistant mutants should serve as useful tools to further study the processes of *Toxoplasma* early invasion, in general, and rhoptry secretion, in particular.

## Introduction

*Toxoplasma gondii* is a widespread, obligate, intracellular parasite able to infect almost any nucleated mammalian and avian cell type. It does this through an active penetration process involving the secretion of discrete secretory organelles: the micronemes, rhoptries and dense granules [1]. Once inside, *Toxoplasma* resides in a parasitophorous vacuole (PV) formed during the process of invasion.

Microneme proteins are involved in gliding motility and the tight association of the parasite with the host cell during early invasion [2]. This is followed by the association of a microneme protein, apical membrane antigen 1 (AMA1), with rhoptry neck proteins (RON2, RON4, RON5, RON8) to form the moving junction (MJ) [3], [4], [5], [6]. Microneme protein 8 (MIC8) has been shown to be required for the secretion of RON4 and therefore necessary to form the MJ [7]. MJ formation is followed by the bulk release of *Toxoplasma* proteins into the host cell at or around the time of invasion. This early release is so far known to consist of several rhoptry proteins, ROP1–4 [8] and ROP18 [9], and the dense granule protein GRA7 [10]. All of these are found in very small, bead-like structures organized in long filamentous strings. These ‘beads-on-a-string’ appear to be associated with the nascent PV [8].

Interestingly, some of these proteins can be found secreted as ‘evacuoles’ into host cells even when parasite invasion is impeded using cytochalasin D, an inhibitor of actin polymerization. This has been observed for ROP1–4 [8] and GRA7 [11]. Some of the proteins present in evacuoles and beads-on-a-string have been shown to also be involved in host-parasite interactions [9], [12], [13].

A second group of *Toxoplasma* proteins have also been shown to be secreted into the host cell. This group of rhoptry proteins comprises a protein phosphatase 2C designated PP2C-hn to reflect its ultimate destination, the host nucleus [14], and a putative protein kinase (ROP16; [15]) that also localizes to the nucleus of infected host cells. PP2C-hn is known to traffic in this way even when parasite invasion is blocked using cytochalasin D [14]. ROP16 is involved in modulating host gene expression following invasion [15].

Virtually nothing is known about the triggers and mechanism of rhoptry secretion or the means by which rhoptry and microneme proteins associate. One model to explain how AMA1 and the RONs interact is that the micronemes fuse to the rhoptry necks. If this were the case, then AMA1 could form a complex with the RONs, which could then be secreted through the rhoptry necks. All host-targeted *Toxoplasma* proteins might then somehow be secreted in bulk by the rhoptries during initial invasion, some as soluble proteins and some in vesicles. This, however, does not explain the ability of micronemal but not rhoptry proteins to be released by extracellular parasites. As to how rhoptry proteins enter the host cell, there is no system known for these parasites that is analogous to type III or type IV secretion system systems found in some bacteria but such a process cannot be excluded.

There are many aspects of *Toxoplasma* early invasion and protein secretion where membrane fusion may be involved. Micronemes may fuse to rhoptries, either or both of these organelles could fuse to the parasite plasma membrane, and the parasite plasma membrane may fuse briefly to the host plasma membranes to allow direct introduction of parasite-derived, exosome-like vesicles. Phospholipase A2s (PLA2s) have been known to be involved in membrane fusion and contribute to membrane fluidity [16], and thus may play a role in *Toxoplasma* invasion and protein secretion.

A number of PLA2 activities have been identified in *Toxoplasma* lysates [17], [18], although no actual genes or proteins have been characterized. Previous research showed that a general, irreversible phospholipase A2 inhibitor, 4-bromophenacyl bromide (4-BPB) blocked parasite invasion into the host cell in a concentration-dependent manner, without metabolically disabling the parasite [19], [20], [21]. When host fibroblasts were pre-incubated with this drug, penetration was not affected [19]. 4-BPB was also shown to inhibit the PLA2 activity from *Toxoplasma* lysates [21]. Addition of exogenous PLA2s was shown to increase invasion and the release of rhoptry proteins into the medium, although the clear possibility that this latter result was due to parasite lysis in the presence of the PLA2 was not excluded [18].

We have used 4-BPB to determine the effect of inhibiting PLA2s on secretion of *Toxoplasma* proteins. We find that 4-BPB specifically blocks rhoptry secretion and invasion but not microneme secretion To gain some clue as to the relevant target of 4-BPB we made mutants resistant to this drug and synthesized ‘click chemistry’ [22], [23] forms of 4-BPB that could be used to covalently tag such proteins. These results are presented below.

## Results

### 4-BPB Treatment Has a Minor Effect on Attachment but Markedly Delays Invasion

To determine the effect of 4-BPB on attachment and invasion of *T. gondii*, we pretreated parasites for 15 minutes with different concentrations of 4-BPB and observed their ability to invade using immunofluorescence assays to detect differential staining with antibodies to the parasite surface. Parasites were treated and then the drug was washed away, since 4-BPB is an irreversible inhibitor.

**Figure 1:**
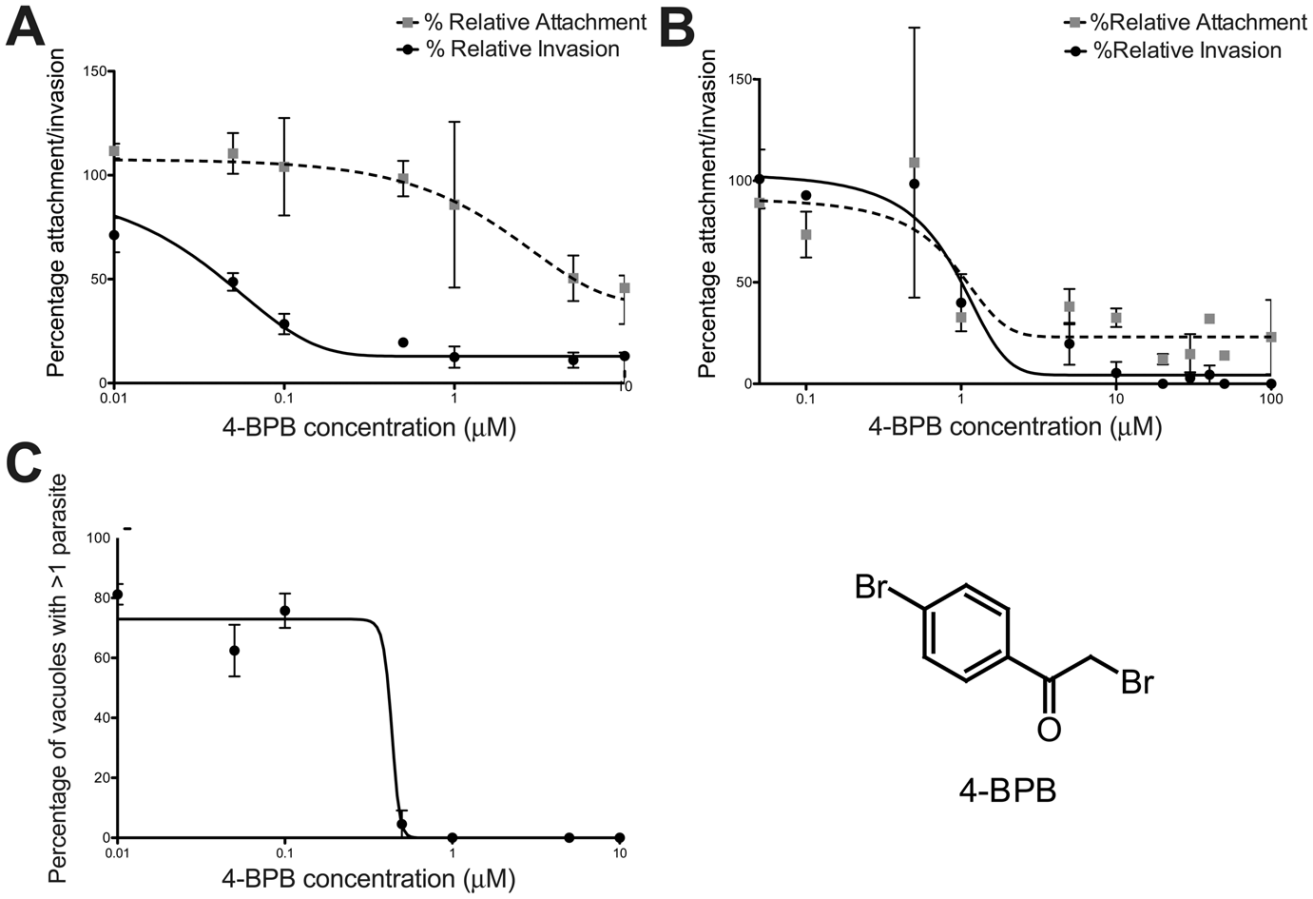
4-BPB inhibits parasite invasion, attachment, and replication. The percentage invasion or attachment of 4-BPB treated parasites was assessed relative to controls. Parasites were added to HFF monolayers under conditions that block invasion (low temperature (**A**) or “Endo” buffer (**B**)). 30 minutes after release of the block (warming to 37°C (**A**) or switching to DMEM (**B**), invasion and attachment were assayed by immunofluorescence staining with antibodies to surface antigen 1 (SAG1). Attachment was distinguished from invasion by sequential staining with mouse- or rabbit-anti-SAG1 before/after permeabilization. Best-fit curves were plotted and the IC50 for invasion was calculated. (**C**) 4-BPB-treated parasites were allowed to infect monolayers for 48 hrs and the percentage of total vacuoles that had more than one parasite was plotted across 4-BPB concentrations. Parasites were observed using anti-SAG1 antibodies in an immunofluorescence assay.

When parasites were given 30 minutes to invade, 4-BPB pretreatment showed a concentration-dependent inhibition of invasion (Figure 1), similar to what was previously reported [19], [20], [21]. These parasites remained attached extracellularly, with most exhibiting protrusion of their conoids (data not shown). 4-BPB pre-treatment resulted in a slight decrease in the efficiency of attachment (Figure 1A, B) but this was considerably less than the effect on invasion. The IC_50_ for 4-BPB-pretreatment was ∼0.022 µM for invasions synchronized by shifting the parasites from a non-permissive to invasion-permissive temperature (Figure 1A). When Endo buffer was used to synchronize invasion, the IC_50_ for inhibiting invasion increased to 0.98 µM, possibly because the parasites showed an increase in attachment efficiency at the same time (Figure 1B). A similar concentration-dependent inhibition of invasion was seen after parasites were given 1 hr and 3 hrs to invade (data not shown).

When parasites were given 6 hrs and longer to invade, 4-BPB-treated parasites were able to invade but failed to replicate. It appeared that the parasites that were previously attached but that had not yet entered the host cell were eventually able to invade, possibly due to turnover of the target of 4-BPB. However, at 4-BPB concentrations greater than 0.5 µM invaded parasites were inhibited in replication and remained permanently in single-parasite vacuoles even when they were given 48 hrs to invade and replicate (Figure 1C) and failed to lyse out of their host cell after 2 weeks (data not shown).

### 4-BPB Inhibits Parasite Motility but Not Microneme Secretion

**Figure 2:**
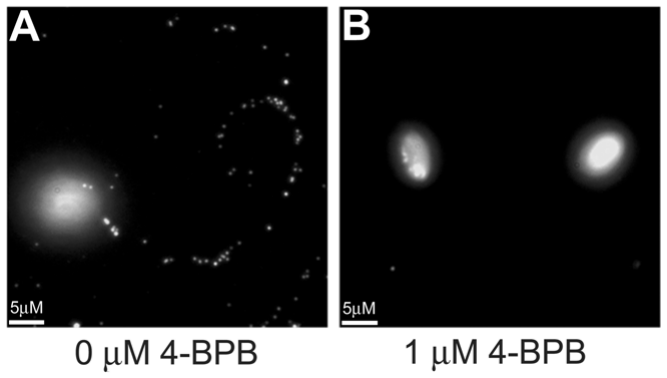
4-BPB inhibits parasite gliding motility. Parasite gliding motility was indirectly assayed by looking for their ability to lay down surface antigen 1 (SAG1) trails on coverslips coated with 5% fetal bovine serum (FBS). Parasites were released from Endo buffer motility block, exposed to medium (A) or 1 µM 4-BPB (B), and allowed to move for 5 minutes prior to fixation. Fixed cells were incubated with anti-SAG1 antibody to visualize SAG1 trails in an immunofluorescence assay. Parasites were in a slightly different focal plane than the trails and as a result are out of focus.

To assess the effects of 4-BPB on motility, we examined the number and length of Surface Antigen 1 (SAG1) ‘gliding trails’ deposited by parasites pretreated with 4-BPB as they migrated on glass coverslips. Trails were visible for concentrations up to 0.5 µM. However, we saw virtually no trails deposited at 4-BPB concentrations of 1 µM and greater (Figure 2). Thus it seems that 4-BPB is able to block gliding motility. These results contrast with previous reports where 4-BPB was seen to have no effect on gliding [19]. Presumably, this reflects a difference in the concentration of active drug used in the two sets of experiments. To determine if the inhibition of gliding motility might be due to a block in microneme secretion, we looked at the secretion of microneme protein 2 (MIC2) both constitutively and in the presence of 1% ethanol and ionophore A23187. Using immunoblot analysis of MIC2 release, we saw no effect on microneme secretion at concentrations up to 25 µM (data not shown) which is much greater than both the IC_50_ for invasion (0.022 µM) and the concentrations at which we saw inhibition of motility (1 µM). There was a slight inhibition of ethanol-induced MIC2 secretion starting at higher concentrations (>30 µM) and leading to a complete inhibition of microneme secretion by 50 µM, probably due to off-target effects. We also looked at the ability of microneme proteins AMA1 and MIC2 to localize to the surface of 4-BPB-pretreated parasites using immunofluorescence and saw no noticeable difference from untreated parasites even at concentrations up to 50 µM (data not shown).

### 4-BPB Treated Parasites Do Not Appear to Secrete Rhoptry Proteins into Evacuoles or Host Nuclei

We looked at the ability of 4-BPB pretreated parasites to secrete rhoptry proteins by immunofluorescence assay. Upon treatment with 4-BPB, uninvaded parasites showed rhoptry protein 1 (ROP1) only in their rhoptries, and not in evacuoles (Figure S1). We also looked by antibody-staining at the ability of 4-BPB-treated parasites to secrete the rhoptry proteins PP2C-hn and ROP16 into the host cells (wherein these proteins normally migrate to the host cell nucleus). No PP2C-hn or ROP16 was detectable anywhere except for the rhoptries of uninvaded 4-BPB treated parasites (data not shown). In addition we looked for the downstream effect of ROP16, the localization of phosphorylated signal transducer and activator of transcription 3 (STAT3) and STAT6 in the nuclei of host cells, and failed to see this when parasites were pretreated with 4-BPB. It should be noted that when parasites eventually invade after 6 hrs, this was accompanied by rhoptry secretion into evacuoles and host nuclei (data not shown).

**Figure 3:**
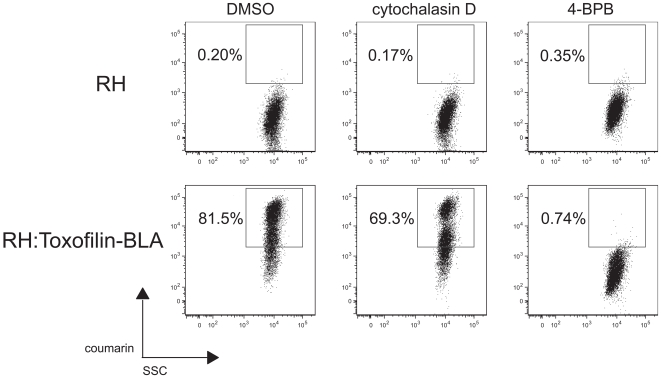
4-BPB inhibits secretion of a toxofilin-beta-lactamase fusion whereas cytochalasin D does not. HFF cells were infected with RH or RH:toxofilin-BLA parasites pretreated with either DMSO, 10 µM 4-BPB or 1 µM cytochalasin D. Invasion was allowed to proceed for 1 hour followed by loading with the cleavable FRET substrate CCF2-AM for 1 hour. The cells were then washed, trypsinized and examined with a 407 nm krypton laser on a modified LSR II flow cytometer (BD, San Jose, CA) for side scatter (SSC) and for the detection of cleaved coumarin in the 410–450 nm channel (“pacific blue” channel). Percentage of total events that are positive for coumarin are also indicated in each plot.

As a more sensitive assay for secretion of rhoptry proteins into host cells, we used a parasite line where the rhoptry protein toxofilin has been fused to beta-lactamase [24]. The presence of beta-lactamase activity inside the host cell can be readily detected in normal infection using a FRET-based assay involving a substrate for the enzyme (cephalosporin) linking two fluorescent molecules (fluorescein and coumarin) in the compound CCF2-AM. 4-BPB treatment completely blocked all detectable introduction of the toxofilin-beta-lactamase fusion into the host cell (Figure 3). In contrast, cytochalasin-D, which is a known inhibitor of invasion, had no measurable effect in this respect. Hence, 4-BPB appears to block invasion at a step upstream of the effect of cytochalasin-D, i.e., before rhoptry protein release.

### 4-BPB Does Not Inhibit Parasite Egress

Host-cell exit can be artificially triggered by the use of the calcium-ionophore A23187 [25]. When infected cells were treated for 15 mins with up to 50 µM 4- BPB and then exposed to up to 2 µM A23187 for 10 minutes, the efficiency and speed of egress was not significantly different from that seen in untreated cultures (data not shown).

### 4-BPB Treated Parasites Fail to Secrete RON4 and Form a Moving Junction

**Figure 4:**
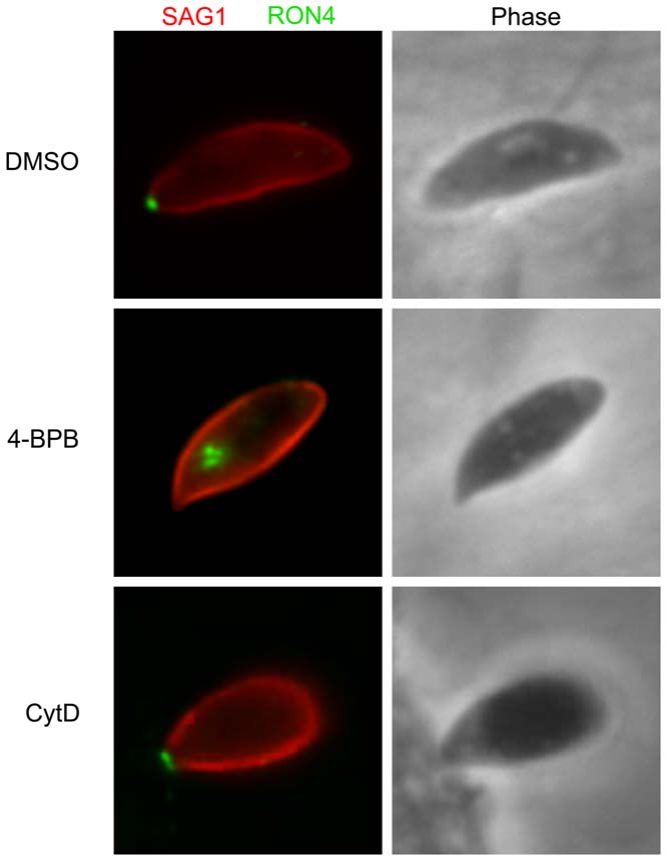
4-BPB blocks secretion of RON4 out of the rhoptry necks. HFF monolayers were infected with parasites pre-treated with either DMSO, 10 µM 4-BPB or 1 µM cytochalasin D. Invasion was allowed to proceed for 3 minutes after which cells were fixed and visualized by immunofluorescence microscopy. An anti-SAG1 antibody was used to stain the surface of extracellular parasites, followed by permeabilization and staining with an anti-RON4 antibody. The corresponding phase microscopy image is included to the right of each fluorescence image.

The above assays concerned introduction of proteins that originate in the rhoptry bulbs and are injected into the host cell. Rhoptry neck proteins (RONs) are released at the earliest points of invasion and, in collaboration with AMA1, form the circular ring of contact between the parasite and host plasma membranes known as the “moving junction” [3], [5]. To determine if release of these RON proteins is also affected by treatment with 4-BPB, we looked at the ability of treated parasites to secrete RON4. Using immunofluorescence assay, we readily detected RON4 only in the region of the rhoptry necks; parasites were blocked in invasion with their conoids protruded, no moving junctions were observed and no RON4 was seen outside the rhoptries (Figure 4). This is very different from the RON4 localization at the apical tip of the parasite during early invasion in untreated parasites, or when parasite invasion was blocked using cytochalasin D, as has been previously noted [3], [5]. RON4 rhoptry neck staining also remained distinct and apical to rhoptry bulb staining when parasites were co-stained with RON4 and the rhoptry bulb marker ROP1 (Figure S2). In fact, the RON4 localization in 4-BPB treated parasites is similar to what was recently described for Toxoplasma mutants in which expression of the microneme protein MIC8 is depleted [7]. Parasites in which MIC8 was depleted showed no further differences in invasion efficiency when treated with 4-BPB (Markus Meissner, personal communication). This could indicate a link between MIC8 and the target of 4-BPB since if MIC8 acted upstream of the target (perhaps as a surface receptor triggering this cascade) there would be no change in the 4-BPB phenotype. Collectively, the above results indicate 4-BPB blocks the invasion process between release of micronemal proteins, such as AMA1, and subsequent release of rhoptry (neck and bulb) proteins.

### Chemically Modified Forms of 4-BPB Can Be Used to Tag Putative Targets

To begin to determine the relevant target of 4-BPB, two methods were used. The first was to use reactive (“click”) derivatives of 4-BPB that would covalently attach to the drug's target(s) and which could then be used as a tag to isolate those targets [22], [23]. To do this, organic synthesis methods were used to replace the 4-bromo of 4-BPB with a 4-propargyl-oxy group that could be used for ‘click chemistry’. The 4-azido substituted derivate is commercially available and was purchased. We will refer to these compounds as 4-propargyl-oxy (4-PPB) and 4-azido (4-APB). Both 4-PPB and 4-APB showed approximately the same concentration-dependent inhibition of parasite invasion and rhoptry secretion as 4-BPB, indicating that the 4-bromo is not required for the activity of the compound (data not shown).

**Figure 5:**
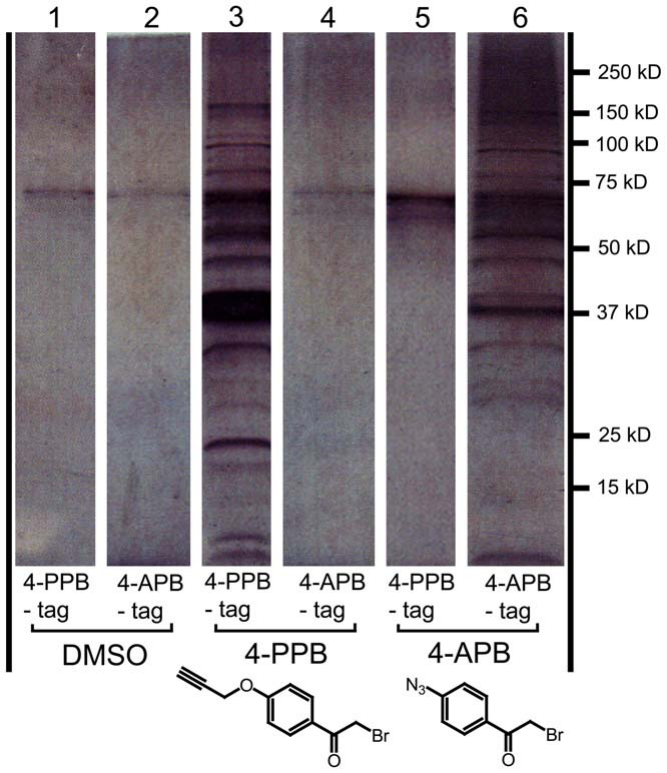
4-BPB tags a large number of proteins by click chemistry. Parasites were treated with either DMSO, 4-PPB or 4-APB and then lysed. The supernatants were then treated with the alkyne- or azide-conjugated biotin-tags, and click chemistry was used to label the targets of the 4-BPB click derivatives. These targets were then purified using streptavidin-conjugated beads and resolved by SDS-PAGE and silver staining. The resulting bands in the silver-stained gel were cut out for identification by LC-MS/MS. Parasites were either treated with DMSO, 0.5 µM 4-PPB or 1 µM 4-APB for 10 minutes, followed by lysis in 1% NP-40 buffer. Each of the lysates was treated with either the 4-APB-tag or the 4-PPB-tag during the click reaction. Lanes are labeled with the inhibitor (DMSO (lanes 1,2), 4-PPB (lanes 3,4), 4-APB (lanes 5,6)) and the click tag (4PPB-tag (lanes 1,3,5) or 4-APB-tag (lanes 2,4,6)) that the corresponding sample was treated with.

The propargyl (alkyne) and azido groups are bioorthogonal reaction partners. That is, they are inert under physiological conditions but can react with each other under special conditions (Cu(I) catalysis). Thus, an alkyne-modified protein can be tagged using an azide-conjugated biotin or, vice versa, an azide-modified protein can react with an alkyne-derivatized biotin [22], [23]. To apply this technique to our system, purified parasites were exposed to either 1 µM 4-APB or 0.5 µM 4-PPB for 10 minutes and then lysed in 1% NP-40 lysis buffer. Next, the click reaction was performed to tag drug-targeted proteins, and the resulting lysates were conjugated to biotin and the tagged proteins purified using Streptavidin beads. These were resolved by SDS-PAGE and silver staining (Figure 5). The results indicated that the click chemistry was highly specific for drug-modified targets, since very few bands were observed when parasites were treated with DMSO or when the wrong tag-inhibitor combination was used. The many bands seen when the correct click tag-inhibitor combination was used and the fact that the patterns were highly similar with both compounds indicates that 4-APB and 4-PPB react with a large number of targets. 4-PPB was also observed to be more reactive than 4-APB for the click reaction, which is typical of the propargyl-containing inhibitor in click reactions.

To determine the identity of the bands tagged with the “click” reagents, mass spectrometry was used. The bands resolved by silver staining were excised and analyzed by LC-MS/MS. While many proteins were thus identified, none were identifiably a phospholipase and none corresponded to a protein whose conjugation to the drug would be expected, *a priori*, to block rhoptry secretion (i.e., none were related to proteins known to be involved in membrane fusion or signaling in other systems) (see Table S1 and Table S2 for a list of the top 100 targets identified using 4-PPB and 4-APB respectively.)

### Generation and Characterization of 4-BPB-Resistant Mutants

To help identify which of the many targets of 4-BPB is key to rhoptry discharge, a genetic approach was taken. Wild-type parasites were treated with the chemical mutagen N-ethyl N-nitrosourea to generate a library of parasites harboring different point mutations. These were then exposed to 4-BPB to select parasites that could grow in the presence of the drug. Unmutagenized parasites were all nonviable after 2 rounds of selection, while rare individuals within the mutagenized populations were able to grow and lyse host cells successfully at concentrations up to 5 µM 4-BPB after 13 rounds of selection. Several clonal lines were obtained from this population of 4-BPB-resistant parasites and all were shown to be able to form plaques at 5 µM 4-BPB, a concentration at which no plaques were formed by wild-type parasites (data not shown). To determine the nature of the resistance to 4-BPB, seven clones were analyzed with respect to mean time for invasion and growth rate once inside the host cells (as described above, 4-BPB both retards invasion and inhibits growth once inside). In all seven cases, there was no significant difference in the speed of invasion compared to wild-type parasites but given enough time, the 4-BPB treated mutants were able to eventually invade, replicate and lyse out of host cells, unlike wild-type parasites (data not shown). Hence, for all seven mutant lines, resistance to the drug appeared to be at the level of not being inhibited once inside the host cell rather than at the level of rhoptry secretion and invasion.

### Mutants Showed No Difference from Wild-Type by Click Chemistry

Despite the fact that the 4-BPB-resistant mutants did not have the desired phenotype in terms of rhoptry discharge and invasion, we sought to determine the identity of the gene in which a mutation had conferred a drug-resistant growth phenotype. To do this, the seven 4-BPB resistant mutants were compared to wild-type parasites by click chemistry, biotin tagging and SDS-PAGE. The results showed no differences in the banding pattern between any of the mutants and wild-type parasites (data not shown). The nature of the difference in the mutants and how that confers 4-BPB-resistance, therefore, remain unknown.

## Discussion

We have shown that 4-BPB is a potent reagent that resolves two previously inseparable aspects of invasion, microneme and rhoptry secretion. This provides new information about the initial secretion events: microneme proteins are able to be secreted onto the parasite surface despite the inhibition of rhoptry secretion. This strongly argues that micronemes do not need to fuse to rhoptry necks to secrete and that formation of the MJ complex, a mix of microneme and rhoptry neck proteins, most likely occurs at the parasite surface. This result complements a recent report showing that two of the MJ proteins, micronemal AMA1 and rhoptry neck protein RON2, form an extremely tight interaction if present in the same compartment but pass through the secretory pathway at different times [4]. These latter authors also argue that the complex forms only once the components reach the surface of the parasite. Interestingly, 4-BPB also blocks invasion by *Plasmodium falciparum* blood stages (Moritz Treeck, personal communication), suggesting that the drug is blocking a common pathway of rhoptry secretion in both parasites.

We have shown that 4-BPB blocks motility of extracellular tachyzoites. Micronemes are known to be involved in parasite gliding motility, but microneme secretion was unaffected at drug concentrations that affected motility. This suggests that the drug affected one or more parasite proteins involved in motility but unrelated to microneme secretion. Since rhoptry discharge has never been linked to motility, the target of 4-BPB that affects motility is likely different from the target affecting rhoptry function. The possibility that rhoptry discharge during invasion is dependent on motility and that just one target of the drug affects both processes seems unlikely as release of rhoptry neck proteins occurs before invasion. The data with the “click” versions of the 4-BPB clearly support the notion that there are multiple targets for this drug.

Despite its ability to inhibit motility, 4-BPB did not also appear to have an effect on parasite egress. When monolayers, containing parasites in intracellular vacuoles, were treated with the drug at concentrations as high as 50 µM, we saw no effect on egress. It is unlikely that the absence of an effect on egress is due to the lack of penetration of the drug, since the drug is predicted to readily enter the host cell and the parasite vacuole. But this possibility cannot be discounted. We could not use concentrations higher than 50 µM, as at those concentrations we started to see toxicity effects on the fibroblasts.

4-BPB is an irreversible inhibitor, and once parasites were pre-treated with it, they were inhibited for invasion for about 6 hrs, despite no drug being present. We also saw no effect when host cells were pre-treated, so the target(s) involved in invasion and rhoptry secretion appear to be parasite-specific. The ability of treated parasites to invade 6 hrs after treatment may be because the relevant target of the drug is turned over in this time period. However, despite being able to invade after 6 hrs, these parasites were still blocked for replication and never formed more than single-parasite vacuoles. They also appeared unable to exit host cells for at least the two weeks that cultures were examined. We were able to get mutants that were resistant to this effect on replication by selecting mutagenized populations with 4-BPB. The fact that these parasites were still blocked in early invasion and rhoptry secretion, however, argues that the mutations were likely in a drug target unrelated to invasion and secretion. Interestingly, the parasites seem to be more easily selected for resistance to the replication target, perhaps because resistance to this represents a stronger selective pressure on the parasites than a block in early invasion and rhoptry secretion. This is consistent with the fact that the effect on invasion is a retardation rather than the absolute block in replication.

Our results indicate that 4-BPB has many more parasite targets than previous work had suggested. Our MS analysis failed to identify candidate PLA2s among them. This may be because PLA2s are not well conserved between species, and consist of a very large class of enzymes [26], [27]; as a result, there may be PLA2s among the identified proteins that are not recognizably such by BLAST analysis. The lack of candidate PLA2s among 4-BPB's putative targets may also be because, based on its chemical structure, 4-BPB is predicted to react with any accessible cysteine or histidine. Thus it could have a substantially broader class of targets than PLA2s alone, and the target responsible for the effects we observed may not be a PLA2.

Despite these limitations, our results show that 4-BPB can be used to specifically uncouple rhoptry and microneme secretion. The drug affects parasite motility and invasion, but not attachment or egress. Hence, 4-BPB, the modified ‘click chemistry’ forms of it, and the resistant mutants, represent useful tools to further study these processes.

## Materials and Methods

### Parasite and Host Cell Culture

*Toxoplasma gondii* tachyzoites of the RH strain were maintained by serial passage in HFF monolayers. HFFs were grown in Dulbecco's modified Eagle's medium (DMEM) (Invitrogen, Carlsbad, CA) supplemented with 10% heat-inactivated fetal calf serum (FCS) (Invitrogen, Carlsbad, CA), 2 mM glutamine, 100 U/ml penicillin, and 100 µg/ml streptomycin.

### Antibodies

The following monoclonal antibodies were used: DG52 (anti-SAG1); Cl22 (anti-AMA1; Tg49 (anti-ROP1), a generous gift from Joseph Schwartzman); 6D10 (anti-MIC2) was provided by Vern Carruthers; and 9D6 (anti-PP2C-hn) was from Peter Bradley. Rabbit antibodies to RON4 (Alexander *et al.* 2005) and SAG1 (provided by Lloyd Kasper) were also used. 3F10, an anti-Hemagglutinin (anti-HA) antibody was obtained from Roche (Palo Alto, CA), and antibodies specific for the phosphorylated forms of STAT3 (phospho-Tyr705) and STAT6 (phospho-Tyr641) from Cell Signaling Technologies, (Danvers, MA). Fluorescent secondary antibodies (Invitrogen/Molecular Probes, Carlsbad, CA) and Hoechst dye (Polysciences, Inc., Warrington, PA) were also used.

### Chemical Handling and Synthesis

Cytochalasin D (Sigma) [28] and A23187 (Calbiochem) [29] were used as previously described. 4-bromophenacyl bromide (4-BPB) and 4-azidophenacyl bromide (4-APB) were obtained from Fluka. These chemicals were highly sensitive to moisture and lost potency if stored in solution. As a result they were stored as dry powder at room temperature in the presence of a dessicant. Working stocks of appropriate concentrations were created fresh in dimethyl sulfoxide (DMSO) and used within 0.5 hours.

### Synthesis of 4-PPB

**Figure 6:**
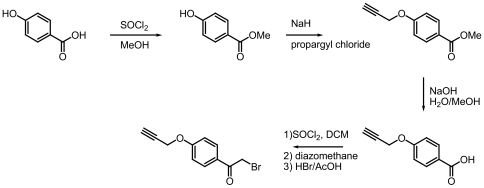
Synthesis of 4-PPB. The synthesis of 4-propargyl-oxy-phenacylbromide (4-PPB) starting with 4-hydroxybenzoic acid is schematically presented. For details, see Materials and Methods.

The compound 4-propargyl-oxyphenacyl bromide (4-PPB) was synthesized according to the route depicted in Figure 6. In brief, 4-hydroxybenzoic acid (Sigma) was esterified under the influence of thionylchloride (1.1 equivalent) and methanol (0.2 M). After completion of the reaction, as monitored by thin-layer chromatography (TLC) analysis, the solvent was removed by evaporation under reduced pressure. To get rid of traces of acid, the residue was taken up in Ethyl acetate (EtOAc), subsequently washed with saturated sodium bicarbonate and brine, dried (magnesium sulfate), filtered and concentrated under reduced pressure. Next, the hydroxyl group was converted into a propargyl ether using sodium hydride and propargyl chloride. Briefly, sodium hydride (1.1 equivalent) was added to a solution of the starting material in N,N-dimethylformamide (DMF; 0.2 M concentration) cooled in an ice bath. After ∼15 minutes, propargyl chloride (1.1 equivalent) was added and reaction progress was monitored by TLC analysis. After completion of the reaction, several drops of methanol were added to quench the excess base. The reaction mixture was evaporated to dryness under reduced pressure. The residue was taken up in EtOAc and washed with saturated sodium bicarbonate and brine, dried (magnesium sulfate), filtered and concentrated under reduced pressure. This was followed by purification by column chromatography. Next, the methyl ester was saponified with sodium hydroxide. The starting material was dissolved in 100% methanol (cooled in ice) and 1.1 equivalent of a 1 M sodium hydroxide solution was added dropwise. The reaction mixture was brought to room temperature and stirred until complete (as determined by TLC analysis). Most of the methanol was evaporated, and EtOAc was added to the residue. The water layer was acidified with 1N HCl, and extracted twice with EtOAc. Combined EtOAc layers were washed with brine, dried (magnesium sulfate), filtered and concentrated. The bromomethyl ketone function was introduced as described previously [30]. Briefly, this involved a three step process: the carboxylic acid was converted to the acid chloride using thionylchloride in dichloromethane and this activated species was reacted with diazomethane to form a diazomethyl ketone, which was converted to the final bromomethyl ketone upon exposure to hydrogen bromide in acetic acid. The final product was purified by column chromatography and analyzed by mass-spectrometry and nuclear magnetic resonance spectroscopy, which confirmed indicated structure.

### Parasite Purification

Monolayers were mechanically lysed by passage through a 27-gauge needle. Large debris was removed by centrifugation at 100×g for 5 min at 10°C. The resulting supernatant was then centrifuged at 300×g for 10 min at 10°C. The 300×g pellet, which contains intact parasites and membranous material from infected HFFs, was resuspended in 2.5 ml of phosphate buffer saline (PBS) and loaded onto a PD-10 desalting column (GE Healthcare, Piscataway, NJ) pre-equilibrated with 30 ml PBS. An additional 3.5 ml of PBS was added to the column, and the displaced buffer was collected as the elution fraction. The elution fraction is enriched for intact parasites while secreted proteins and debris from the infected HFF lysate remain on the column. To avoid clogs, no more than 2.5×10^7^ infected HFF equivalents were added to each column.

### Immunofluorescence

Purified parasites in room-temperature PBS were treated for 15 minutes with different concentrations of freshly prepared 4-BPB or with DMSO as a control. Parasites were spun down and washed twice with PBS, following which HFF monolayers on glass cover slips were infected with parasites at a multiplicity of infection (MOI) of ∼10. To synchronize invasion, parasites were allowed to attach to HFF monolayers seeded on cover slips for 20 minutes at ∼1°C, a temperature that prevents invasion. The cover slips were then shifted to 37°C, an invasion-permissive temperature, for 15 minutes before fixation. Alternatively, invasion was synchronized using Endo buffer (44.7 mM K_2_SO_4_, 10 mM MgSO_4_, 106 mM sucrose, 5 mM glucose, 20 mM Tris–H_2_SO_4_, 3.5 mg/mL BSA, pH 8.2) [31].

Invasion was allowed to proceed for 30 minutes, 1 hr, 3 hrs, 6 hrs, 24 hrs and 48 hrs, after which infected cells were washed thrice in PBS and fixed in PBS plus 4.0% (w/v) formaldehyde for 20 minutes. The cover slips were washed in PBS and blocked in PBS plus 3% (w/v) bovine serum albumin (BSA) for 2 hrs. After staining with rabbit anti-SAG1 antibody, monolayers were permeabilized using PBS containing 3% BSA and 0.2% (v/v) Triton X-100 and then incubated with mouse anti-SAG1 antibody. Parasites staining with rabbit-anti-SAG1 were scored as extracellular, while parasites staining only with mouse-anti-SAG1 were scored as intracellular.

To assay for the ability of microneme proteins to get to the parasite surface, parasites on coverslips coated with 5% FBS were fixed after 30 minutes. They were then stained with anti-SAG1 antibodies, as well as either anti-AMA1 or anti-MIC2 antibodies without permeabilization.

To detect rhoptry secretion, parasites were allowed to invade for 30 minutes or 1 hr and stained with anti-SAG1 antibody, then permeabilized and stained with either anti-ROP1 or anti-PP2C-hn antibodies. Analysis of evacuoles was performed as described previously [28]. An anti-HA antibody was also used to detect a C-terminally HA-tagged ROP16 fusion protein secreted by parasites (described in [15]). Antibodies to phopho-STAT3 and phospho-STAT6 were also used to assay for signaling downstream of ROP16 secretion as described previously [15].

To detect secretion of rhoptry neck proteins and moving junction formation, parasites were allowed to invade for 3 minutes after shifting them to invasion-permissive temperature, and after fixation and staining with anti-SAG1 antibody, were permeabilized and then stained with anti-RON4 antibody. Parasites were also treated with 1 µM cytochalasin D and allowed to invade as above to contrast RON4 secretion during inhibition of invasion by cytochalasin D with inhibition by 4-BPB.

Gliding motility was assessed indirectly by immunofluorescence analysis of SAG1 in trails [32]. After treatment with 4-BPB, parasites were immobilized in Endo buffer; [33]) and were seeded onto coverslips that had been coated overnight with 5% fetal bovine serum. After 20 min at 37°C, the K_2_SO_4_ buffer was removed and replaced with DMEM and the parasites were incubated for 5 min more at 37°C. The parasites were then fixed as described before and stained using anti-SAG1 antibody.

Fixed cells were incubated for an hour with primary antibodies diluted in PBS/3%BSA/0.01% Triton X-100. After 3–5 washes in PBS, the samples were incubated for one hour with secondary antibodies diluted in PBS plus 3% BSA. After 3–5 washes in PBS, samples were mounted in Vectashield (Vector Laboratories, Burlingame, CA) on microscope slides.

Phase and fluorescence images were captured on a Hamamatsu Orca100 CCD camera coupled to an Olympus BX60 microscope and were processed using Image-Pro Plus 2.0 (MediaCybernetics, Silver Spring, MD) and Photoshop CS4 (Adobe Systems, San Jose, CA). Image processing was limited to cropping, pseudo-coloring, merging of individual color channels into single images, and adjusting of tonal ranges to reproduce observations faithfully.

### 4-BPB Titration

Data was collected from 10 fields per coverslip, for each 4-BPB concentration from independent experiments. The number of parasites staining either only with mouse-anti-SAG1 (intracellular) or only with rabbit-anti-SAG1 (extracellular) were counted and this number was used to calculate the percentage of invaded parasites out of the total staining with either antibody. The total number of parasites per field at each concentration of 4-BPB was used as a measure of parasite attachment. Percentage invasion and attachment at each concentration relative to the DMSO-treated control parasites was calculated. At time-points of invasion 6 hrs and longer, the number of vacuoles containing a single parasite versus multiple parasites was also counted, in order to calculate the percentage of the total vacuoles that had more than one parasite. The data was plotted using GraphPad Prism (GraphPad Software, La Jolla, CA) and the four parameter logistic model was used to calculate the IC_50_ of 4-BPB for invasion and attachment.

### β-Lactamase Assay

This assay was originally described for assessing introduction of bacterial effector proteins [34] based on a mammalian reporter system [35]. It has been adapted for use in *Toxoplasma* [24]. Briefly, for visualization of β-lactamase (BLA) activity, RH or toxofilin-BLA parasites were treated with DMSO, 1 µM cytochalasin D, or 0.5, 5 or 50 µM 4-BPB for 10 minutes. Monolayers of HFF cells grown on glass chamber slides were infected with the treated parasites at a nominal MOI of 10 and incubated at 37°C. At various times post-infection, the infected HFF were incubated with the BLA substrate CCF2-AM (Invitrogen, Carlsbad, CA) at a 1X concentration in complete DMEM for 1 hour at room temperature in the dark. Live, infected cells were then visualized using a Leica SP2 AOBS Confocal Laser Scanning Microscope (Cell Sciences Imaging Facility, Stanford University, Stanford, CA) with a blue diode 405 nm laser for excitation and with detection filters set at 410–450 nm for coumarin and 493–550 nm for fluorescein.

For flow cytometry, RH or toxofilin-BLA parasites were treated with 1 µM cytochalasin D, 10 µM 4-BPB or with DMSO as a control for 10 minutes and then added to monolayers of HFF in 6-well dishes at a nominal MOI of 10. The infected cells were incubated at 37°C for 1 hour and loaded with the BLA substrate CCF2-AM at a 1X concentration for 1 hour at room temperature in the dark. The cells were washed with 1X PBS and trypsinized. The resuspended cells were examined on a modified LSR II flow cytometer (BD, San Jose, CA) with the 407 nm krypton laser for the detection of coumarin (in the pacific blue channel).

### Egress

Egress assays were performed as previously described [36]. 2 µM A23187 was used for 10 mins and results were observed by phase microscopy.

### Microneme Secretion

For preparation of excretory-secretory antigens (ESA), approximately 10^8^ tachyzoites were washed, treated with 4-BPB as described above, and resuspended in 1 ml of DMEM containing 20 mM Hepes and 1% FBS. They were then stimulated to discharge micronemes either by addition of calcium ionophore A23187 to a final concentration of 2 µM or ethanol to a final concentration of 1.0%, and incubated at 37°C for 30 minutes, as described previously [29]. Cells were removed by centrifugation at 2000×g and the supernatant and pellet were analyzed on immunoblot.

### Western Blot Analysis

Protein samples were separated by SDS-PAGE and transferred to nitrocellulose membranes. Typically, membranes were blocked for three hours in PBS-T (PBS, 0.1% Tween-20) containing 5% (w/v) milk, incubated for one hour with primary antibodies, washed thrice with PBS-T, incubated for one hour with secondary antibodies, and washed thrice with PBS-T. Primary and secondary antibodies were diluted in PBS-T plus 1% (w/v) milk. Horseradish peroxidase-conjugated goat anti-rabbit and goat anti-mouse antibodies (Bio-Rad, Hercules, CA) were used as secondary antibodies. Horseradish peroxidase activity was visualized using the SuperSignal West Pico Chemiluminescent Substrate (Pierce, Rockford, IL).

### Protein Labeling and Click Chemistry

Parasites were purified as described above and treated either with DMSO, 4-PPB or 4-APB at different concentrations for 15 minutes at room temperature. Parasites were spun down and washed three times in PBS, and resuspended in 1% NP-40 lysis buffer containing Complete (Roche) protease inhibitors. After being left on ice for 2 hrs, parasites were spun down for 10minutes at 1000×g at 4°C, and the supernatant was used for the click reaction.

Click chemistry was performed as described previously [22], [23]. Briefly, 50 µM of the alkyne- or azide-conjugated biotin-tags (50X stock in DMSO), 1 mM tris (2-carboxyethyl)phosphine (TCEP from Sigma; fresh 50X stock in water), 100 µM tris-(benzyltriazolylmethyl)amine (TBTA) ligand (17X stock in DMSO:t-Butanol 1∶4) and 1 mM copper sulfate (CuSO_4_; 50x stock in water) were added to each sample. These were then allowed to react for 1 hr at room temperature with rocking. Samples were then either mixed with sample buffer for SDS-PAGE and subsequent treatment with streptavidin-HRP (Sigma), or used directly for precipitation.

For precipitations, the samples were incubated for 2 hrs with UltraLink streptavidin beads (Pierce) that had been pre-washed in PBS. The beads were then spun down and washed three times each with 0.05% sodium dodecyl sulfate (SDS), 1 M sodium chloride, 10% ethanol, and once with PBS. After spinning down the beads and removing the supernatant, the beads were resuspended in sample buffer, vortexed and boiled for 5 minutes. Following this the samples were analyzed by SDS-PAGE in precast 12% protein gels followed by staining with silver stain plus (Bio-Rad). Bands were cut out and sent for analysis by LC-MS/MS.

### LC-MS/MS Analysis

The samples were analyzed using a Thermo Fisher Scientific LTQ FTMS mass spectrometer, (San Jose, California). The precursor survey scan was carried out on the FTMS with 100,000 resolution, using a scan range of 400–1400 m/z. Five MS/MS scans were performed on the top five peaks following the precursor scan. Data was searched using the data base search engine Mascot, (London, England).

### Mutagenesis

Chemical mutagenesis of RH parasites was done using the standard protocol with N-ethyl N-nitrosourea (ENU; 75 µg ml−1) at 37°C for 1 h [37]. Mutagenized parasites were passaged twice in 175 cm^2^ flasks and split into two independent populations prior to selection. A control population was treated with DMSO instead of ENU.

### Selection

Parasites from the two mutagenized populations were purified and treated with 1 µM 4-BPB for 15 mins as described above. The parasites were spun down and washed three times in PBS before they were allowed to invade fresh HFFs in 75 cm^2^ flasks. Following expansion of these populations, selection with 1 µM 4-BPB was repeated and followed by growing the parasites on 25 cm^2^ flasks. 3 rounds of selection at 1 µM 4-BPB were followed by 5 rounds at 2 µM 4-BPB and another 5 rounds at 5 µM 4-BPB. Parasites that survived these 13 rounds of selection were plated onto monolayers in a 96-well plate at a limiting dilution to isolate single clones. Single clones were screened in a plaque assay for resistance to 5 µM 4-BPB. Seven such mutant clones were isolated, 4 from one independent population and 3 from the other. The plaque assay was performed as described previously [38]. Monolayers of HFF, grown in six-well plates, were infected with 50–250 tachyzoites per well. After 1 week of incubation at normal growth conditions (37°C, 5% CO_2_), cells were fixed for 1 minute with −20°C 100% methanol, stained with crystal violet for 5 minutes and washed once with PBS. Documentation was performed with a Nikon SMZ 1500 binocular at 70-fold magnification.

Mutants were characterized by immunofluorescence assays as described previously, to determine their ability to invade and secrete rhoptries in the presence of 4-BPB. Click chemistry and subsequent analysis by immunoblot to compare the mutant clones to the wild-type parasites was performed as described above.

## Supporting Information

Figure S1 4-BPB inhibits secretion of ROP1 evacuoles - HFF monolayers were infected with parasites pre-treated with either DMSO or 10 µM 4-BPB or 1 µM cytochalasin D. Invasion was allowed to proceed for 3 minutes following which monolayers were fixed and visualized by immunofluorescence microscopy. An anti-SAG1 antibody was used to stain the surface of extracellular parasites, followed by permeabilization and staining with an anti-ROP1 antibody. The corresponding phase microscopy image is included to the right of each fluorescence image. Characteristic intracellular ROP1 evacuoles were observed secreted by DMSO-treated and cytochalasin D treated parasites, but were not observed to be secreted by 4-BPB treated parasites. ROP1 signal from inside the rhoptries is extremely weak since the permeabilization condition used (100% ethanol at room temperature) is not particularly efficient at permeabilizing the rhoptry organelles.
(7.50 MB TIF)

Figure S2 RON4 and ROP1 show distinct localization in 4-BPB treated parasites - HFF monolayers were infected with parasites pre-treated with 10 µM 4-BPB. Invasion was allowed to proceed for 3 minutes following which cells were fixed and visualized by immunofluorescence microscopy. Following permeabilization with 100% ethanol, anti-RON4 and anti-ROP1 antibodies were used to stain the rhoptry neck and rhoptry bulb compartments, respectively. The corresponding phase microscopy image is included to the right of the fluorescence image. RON4 did not colocalize with ROP1 and appeared to show rhoptry neck localization, apical to and quite distinct from ROP1 rhoptry bulb localization.
(1.49 MB TIF)

Table S1 List of top 100 targets identified by LC-MS/MS using 4-PPB, the alkyne click derivative of 4-BPB
(0.13 MB DOC)

Table S2 List of top 100 targets identified by LC-MS/MS using 4-APB, the azide click derivative of 4-BPB
(0.12 MB DOC)
