# Supplementary figures and images for: 4-Bromophenacyl Bromide Specifically Inhibits Rhoptry Secretion during Toxoplasma Invasion
Source: PLoS One. 2009 Dec 2;4(12):e8143. doi: 10.1371/journal.pone.0008143 (PMC2780294; doi:10.1371/journal.pone.0008143)

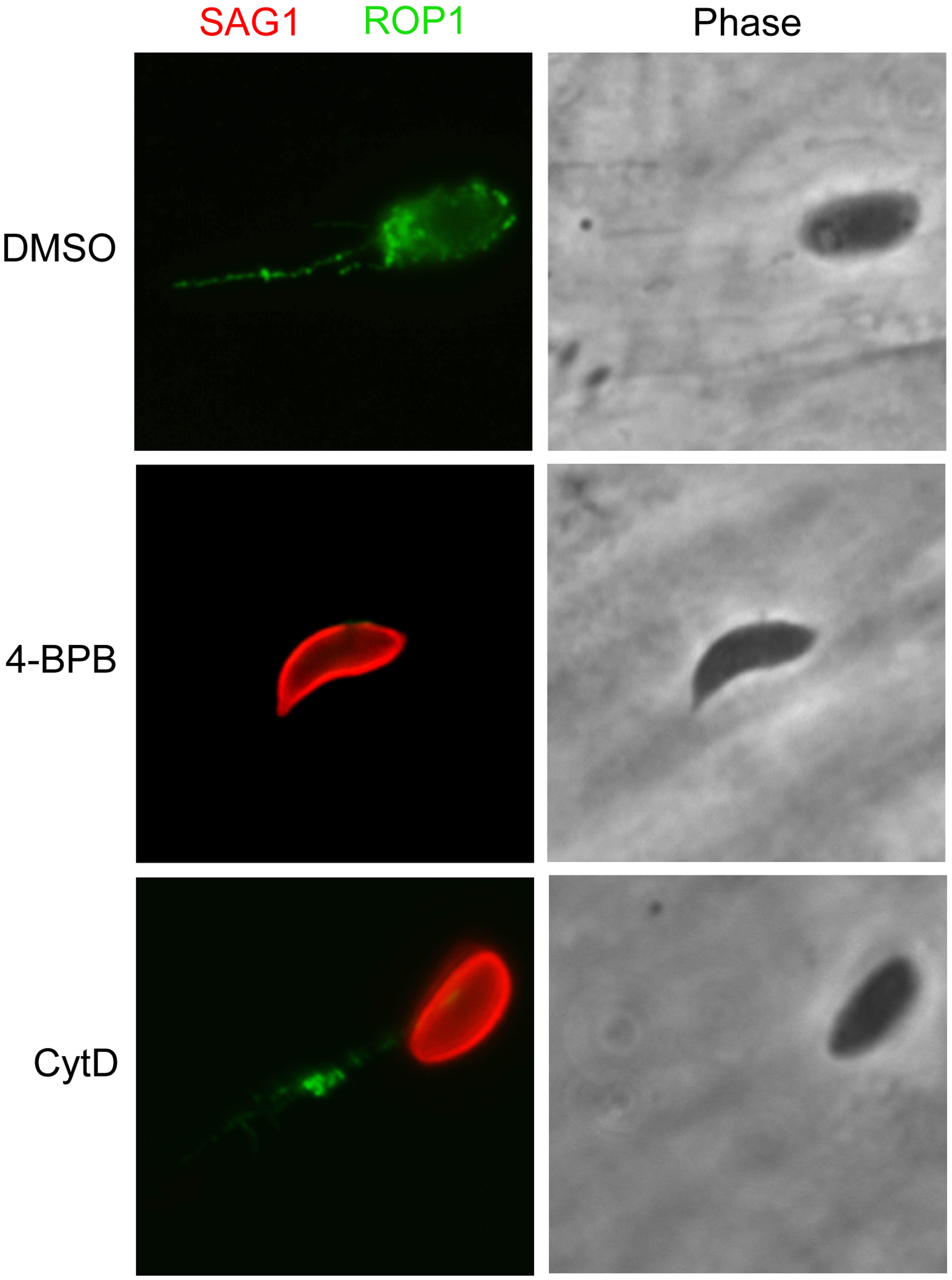

Supplement: Figure S1 — 4-BPB inhibits secretion of ROP1 evacuoles - HFF monolayers were infected with parasites pre-treated with either DMSO or 10 µM 4-BPB or 1 µM cytochalasin D. Invasion was allowed to proceed for 3 minutes following which monolayers were fixed and visualized by immunofluorescence microscopy. An anti-SAG1 antibody was used to stain the surface of extracellular parasites, followed by permeabilization and staining with an anti-ROP1 antibody. The corresponding phase microscopy image is included to the right of each fluorescence image. Characteristic intracellular ROP1 evacuoles were observed secreted by DMSO-treated and cytochalasin D treated parasites, but were not observed to be secreted by 4-BPB treated parasites. ROP1 signal from inside the rhoptries is extremely weak since the permeabilization condition used (100% ethanol at room temperature) is not particularly efficient at permeabilizing the rhoptry organelles. (7.50 MB TIF) [file pone.0008143.s001.tif]

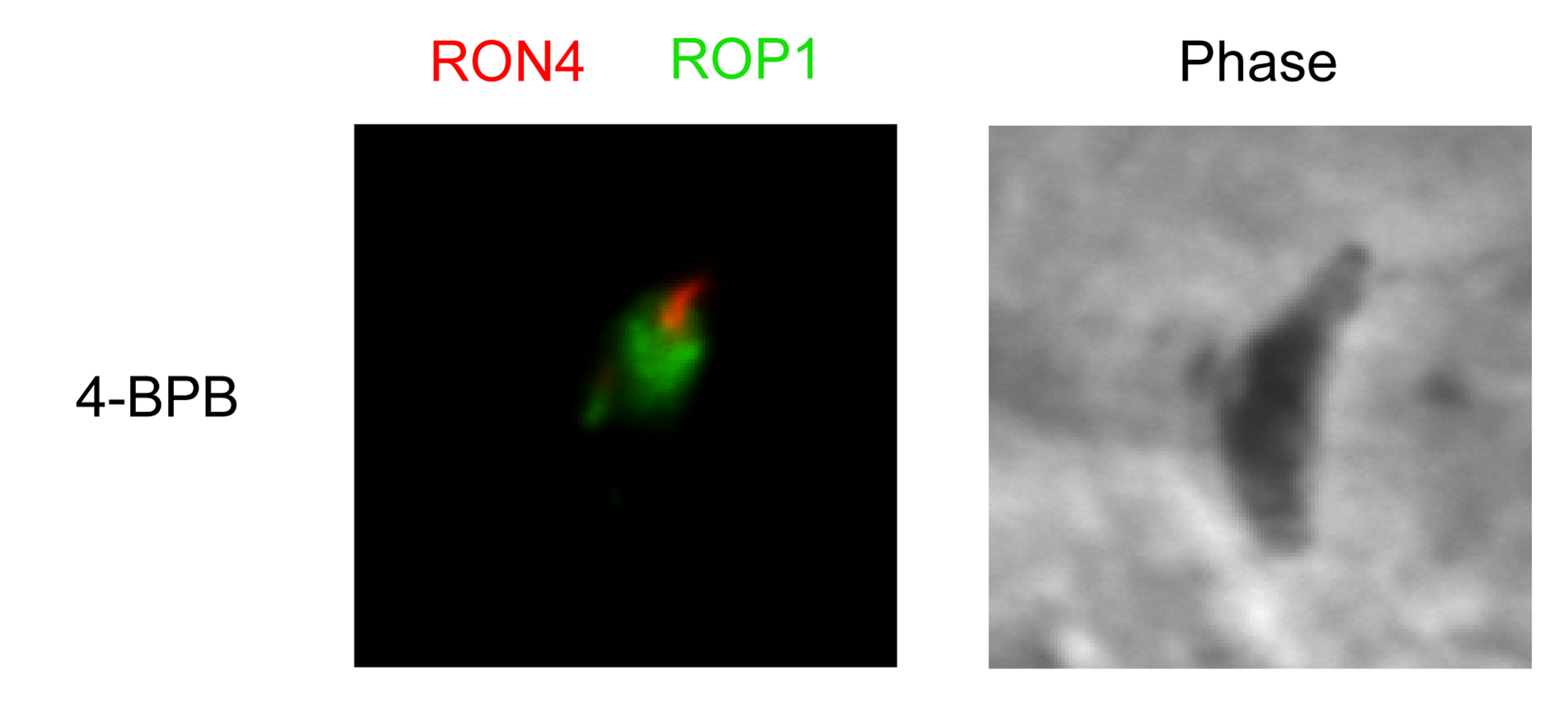

Supplement: Figure S2 — RON4 and ROP1 show distinct localization in 4-BPB treated parasites - HFF monolayers were infected with parasites pre-treated with 10 µM 4-BPB. Invasion was allowed to proceed for 3 minutes following which cells were fixed and visualized by immunofluorescence microscopy. Following permeabilization with 100% ethanol, anti-RON4 and anti-ROP1 antibodies were used to stain the rhoptry neck and rhoptry bulb compartments, respectively. The corresponding phase microscopy image is included to the right of the fluorescence image. RON4 did not colocalize with ROP1 and appeared to show rhoptry neck localization, apical to and quite distinct from ROP1 rhoptry bulb localization. (1.49 MB TIF) [file pone.0008143.s002.tif]
