# Supplementary material for: 4-Bromophenacyl Bromide Specifically Inhibits Rhoptry Secretion during Toxoplasma Invasion
Source: PLoS One. 2009 Dec 2;4(12):e8143. doi: 10.1371/journal.pone.0008143 (PMC2780294; doi:10.1371/journal.pone.0008143)
Supplement: Table S1 — List of top 100 targets identified by LC-MS/MS using 4-PPB, the alkyne click derivative of 4-BPB (0.13 MB DOC) [file pone.0008143.s003.doc]

**SUPPLEMENTARY TABLE 1: List of top 100 targets identified by LC-MS/MS using 4-PPB, the alkyne click derivative of 4-BPB**

| Protein Hit Rank | ToxoDB Gene ID | Protein Description | Number of Assigned Peptide Matches |
| --- | --- | --- | --- |
| 1 | 50.m03211 | ATP-dependent DNA helicase II, 70 kDa subunit, putative | 2829 |
| 2 | 80.m00003 | glyceraldehyde-3-phosphate dehydrogenase | 1717 |
| 3 | 80.m00088 | cytosol aminopeptidase | 1649 |
| 4 | 59.m03410 | enolase, putative | 1476 |
| 5 | 59.m00003 | heat shock protein 70, putative | 1298 |
| 6 | 44.m00006 | lactate dehydrogenase | 728 |
| 7 | 80.m00001 | heat shock protein 90 | 681 |
| 8 | 80.m02365 | hypothetical protein | 663 |
| 9 | 76.m01689 | kinesin central motor, putative | 596 |
| 10 | 59.m03518 | asparaginyl-tRNA synthetase, putative | 460 |
| 11 | 583.m00011 | subtilase family serine protease, putative | 432 |
| 12 | 42.m00123 | phosphofructokinase, putative | 417 |
| 13 | 25.m00007 | actin | 403 |
| 14 | 35.m00882 | proteasome subunit alpha type 3, putative | 394 |
| 15 | 76.m00016 | elongation factor 1-alpha, putative | 392 |
| 16 | 55.m00011 | hydroxymethyldihydropterin pyrophosphokinase-dihydropteroate synthase | 388 |
| 17 | 55.m00015 | 14-3-3 protein, putative | 381 |
| 18 | 46.m00002 | fructose-1,6-bisphosphate aldolase | 373 |
| 19 | 641.m00193 | phosphoglycerate kinase, putative | 353 |
| 20 | 20.m03764 | cell wall protein-related | 345 |
| 21 | 55.m04665 | glycyl-tRNA synthetase, putative | 296 |
| 22 | 80.m02134 | hypothetical protein | 277 |
| 23 | 80.m00063 | tryptophanyl-tRNA synthetase, putative | 271 |
| 24 | 20.m03912 | elongation factor 2, putative | 262 |
| 25 | 49.m03373 | haloacid dehalogenase-like hydrolase domain-containing protein | 258 |
| 26 | 44.m00037 | serine-threonine phosophatase 2C | 246 |
| 27 | 20.m03918 | nascent polypeptide-associated complex alpha chain, putative | 237 |
| 28 | 55.m00007 | pyruvate kinase, putative | 232 |
| 29 | 50.m00020 | seryl-tRNA synthetase, putative | 170 |
| 30 | 44.m00052 | 58 kDa phosphoprotein, putative | 168 |
| 31 | 39.m00001 | actin depolymerizing factor | 167 |
| 32 | 83.m01195 | NADP-specific glutamate dehydrogenase, putative | 165 |
| 33 | 583.m05344 | conserved hypothetical protein | 165 |
| 34 | 38.m01113 | heat shock protein, putative | 159 |
| 35 | 583.m00630 | purine nucleoside phosphorylase, putative | 159 |
| 36 | 20.m03930 | 20k cyclophilin | 158 |
| 37 | 44.m04669 | 40S ribosomal protein S3, putative | 149 |
| 38 | 44.m00005 | peroxisomal catalase | 147 |
| 39 | 583.m00642 | hypothetical protein | 132 |
| 40 | 641.m00168 | malate dehydrogenase, putative | 131 |
| 41 | 59.m03592 | inhibitor-1 of protein phosphatase type 2A | 128 |
| 42 | 27.m00003 | protein disulfide isomerase, putative | 120 |
| 43 | 42.m00050 | triosephosphate isomerase, putative | 118 |
| 44 | 42.m00069 | elongation factor 1-beta, putative | 112 |
| 45 | 129.m00257 | conserved hypothetical protein | 111 |
| 46 | 33.m01287 | peptidase M16 inactive domain-containing protein / insulinase (peptidase family M16) domain-containing protein | 111 |
| 47 | 49.m03152 | proteasome subunit alpha type 4, subunit | 111 |
| 48 | 44.m04694 | kelch motif domain-containing protein | 110 |
| 49 | 50.m03396 | eukaryotic translation initiation factor 3 subunit 3, putative | 106 |
| 50 | 48.m00179 | hypothetical protein | 103 |
| 51 | 52.m02694 | hypothetical protein | 103 |
| 52 | 162.m00001 | calcium-dependent protein kinase, putative / calmodulin-domain protein kinase, putative | 101 |
| 53 | 41.m00030 | serine/threonine protein phosphatase, putative | 99 |
| 54 | 27.m00845 | hypothetical protein | 97 |
| 55 | 80.m00002 | phosphoenolpyruvate carboxykinase, putative | 96 |
| 56 | 52.m00018 | hypothetical protein | 94 |
| 57 | 50.m03331 | hypothetical protein | 87 |
| 58 | 55.m08214 | nucleosome assembly protein-related | 85 |
| 59 | 49.m03383 | hypothetical protein | 80 |
| 60 | 69.m00139 | conserved hypothetical protein | 79 |
| 61 | 38.m01889 | vacuolar ATP synthase subunit B, putative | 78 |
| 62 | 49.m03357 | HIT domain-containing protein | 78 |
| 63 | 55.m00081 | proteasome subunit alpha type 7, putative | 77 |
| 64 | 57.m00025 | 40S ribosomal protein SA, putative | 76 |
| 65 | 25.m01794 | hypothetical protein | 75 |
| 66 | 33.m01348 | aggrecan core protein-related | 75 |
| 67 | 65.m00014 | proteasome subunit alpha type 1, putative | 75 |
| 68 | 80.m00014 | nucleosome assembly domain-containing protein | 73 |
| 69 | 72.m00001 | non-transmembrane antigen | 73 |
| 70 | 113.m00807 | aspartyl aminopeptidase, putative | 70 |
| 71 | 59.m03661 | cell division protein 48, putative | 70 |
| 72 | 33.m02668 | intracellular protease, putative | 67 |
| 73 | 583.m00606 | hypothetical protein | 66 |
| 74 | 37.m00743 | glutaminyl-tRNA synthetase, putative | 65 |
| 75 | 72.m00688 | proteasome A-type and B-type domain-containing protein | 64 |
| 76 | 46.m01675 | dynein 1-beta heavy chain, flagellar inner arm I1 complex, putative | 63 |
| 77 | 145.m00604 | threonyl-tRNA synthetase, putative | 63 |
| 78 | 38.m00011 | phosphoserine aminotransferase, putative | 63 |
| 79 | 55.m08205 | X-prolyl aminopeptidase, putative | 62 |
| 80 | 583.m05329 | long-chain-fatty-acid--CoA ligase, putative | 62 |
| 81 | 55.m04872 | 60S acidic ribosomal protein P1, putative | 61 |
| 82 | 50.m03413 | proteasome activator subunit, putative | 61 |
| 83 | 49.m00047 | TCP-1/cpn60 family chaperonin, putative | 61 |
| 84 | 583.m05601 | hypothetical protein | 59 |
| 85 | 44.m04672 | sepiapterin reductase, putative | 57 |
| 86 | 641.m01538 | ubiquitin-conjugating enzyme E2, putative | 56 |
| 87 | 42.m03585 | hypothetical protein | 56 |
| 88 | 65.m01101 | SNF2 family helicase, putative | 56 |
| 89 | 76.m00019 | MA3 domain protein | 56 |
| 90 | 113.m00016 | 2,3-bisphosphoglycerate-dependent phosphoglycerate mutase, (phosphoglycerate mutase), putative | 54 |
| 91 | 72.m00843 | KH domain containing protein | 53 |
| 92 | 44.m02718 | protein phosphatase 2C, putative | 51 |
| 93 | 69.m00140 | proliferation-associated protein 2G4, putative | 51 |
| 94 | 49.m03356 | 40S ribosomal protein S26, putative | 51 |
| 95 | 50.m05680 | eukaryotic translation initiation factor 4A | 51 |
| 96 | 80.m02344 | HECT-domain (ubiquitin-transferase) containing protein | 50 |
| 97 | 25.m01849 | anti-silencing protein 1, putative | 50 |
| 98 | 50.m03346 | tetrapyrrole (corrin/porphyrin) methylase domain-containing protein | 50 |
| 99 | 44.m02617 | dynein heavy chain, putative | 49 |
| 100 | 59.m07805 | PWI domain containing protein | 49 |
