# Supplementary material for: 4-Bromophenacyl Bromide Specifically Inhibits Rhoptry Secretion during Toxoplasma Invasion
Source: PLoS One. 2009 Dec 2;4(12):e8143. doi: 10.1371/journal.pone.0008143 (PMC2780294; doi:10.1371/journal.pone.0008143)
Supplement: Table S2 — List of top 100 targets identified by LC-MS/MS using 4-APB, the azide click derivative of 4-BPB (0.12 MB DOC) [file pone.0008143.s004.doc]

**SUPPLEMENTARY TABLE 2: List of top 100 targets identified by LC-MS/MS using 4-APB, the azide click derivative of 4-BPB**

| Protein Hit Rank | ToxoDB Gene ID | Protein Description | Number of Assigned Peptide Matches |
| --- | --- | --- | --- |
| 1 | 80.m00003 | glyceraldehyde-3-phosphate dehydrogenase | 7483 |
| 2 | 80.m00088 | cytosol aminopeptidase | 3373 |
| 3 | 50.m03211 | ATP-dependent DNA helicase II, 70 kDa subunit, putative | 2530 |
| 4 | 44.m00005 | peroxisomal catalase | 2303 |
| 5 | 59.m03410 | enolase, putative | 2032 |
| 6 | 27.m00003 | protein disulfide isomerase, putative | 1828 |
| 7 | 59.m03518 | asparaginyl-tRNA synthetase, putative | 1477 |
| 8 | 25.m00007 | actin | 1422 |
| 9 | 59.m00003 | heat shock protein 70, putative | 1339 |
| 10 | 55.m00011 | hydroxymethyldihydropterin pyrophosphokinase-dihydropteroate synthase | 946 |
| 11 | 80.m00001 | heat shock protein 90 | 908 |
| 12 | 20.m03918 | nascent polypeptide-associated complex alpha chain, putative | 837 |
| 13 | 80.m00063 | tryptophanyl-tRNA synthetase, putative | 802 |
| 14 | 55.m00015 | 14-3-3 protein, putative | 797 |
| 15 | 59.m06060 | la domain-containing protein | 762 |
| 16 | 44.m00006 | lactate dehydrogenase | 699 |
| 17 | 583.m00630 | purine nucleoside phosphorylase, putative | 691 |
| 18 | 55.m04635 | pyridine nucleotide-disulphide oxidoreductase, putative | 640 |
| 19 | 55.m04635 | phosphofructokinase, putative | 595 |
| 20 | 76.m00016 | elongation factor 1-alpha, putative | 591 |
| 21 | 80.m02365 | hypothetical protein | 527 |
| 22 | 20.m03912 | elongation factor 2, putative | 523 |
| 23 | 25.m02949 | hypothetical protein | 522 |
| 24 | 59.m03411 | enolase, putative | 503 |
| 25 | 583.m05668 | hypothetical protein | 498 |
| 26 | 35.m00882 | proteasome subunit alpha type 3, putative | 490 |
| 27 | 44.m02718 | protein phosphatase 2C, putative | 477 |
| 28 | 83.m01295 | N-glycosylase/DNA lyase-related | 467 |
| 29 | 46.m00002 | fructose-1,6-bisphosphate aldolase | 464 |
| 30 | 38.m01067 | alanyl-tRNA synthetase, putative | 456 |
| 31 | 583.m00002 | peroxidoxin 2 | 440 |
| 32 | 76.m01689 | kinesin central motor, putative | 437 |
| 33 | 583.m00011 | subtilase family serine protease, putative | 430 |
| 34 | 33.m02668 | intracellular protease, putative | 412 |
| 35 | 59.m03592 | inhibitor-1 of protein phosphatase type 2A | 407 |
| 36 | 55.m08214 | nucleosome assembly protein-related | 405 |
| 37 | 44.m02770 | deoxyuridine 5~-triphosphate nucleotidohydrolase, putative | 372 |
| 38 | 50.m05680 | eukaryotic translation initiation factor 4A | 367 |
| 39 | 50.m00020 | seryl-tRNA synthetase, putative | 365 |
| 40 | 55.m04665 | glycyl-tRNA synthetase, putative | 355 |
| 41 | 76.m01640 | prolyl endopeptidase, putative | 348 |
| 42 | 145.m00322 | cysteinyl-tRNA synthetase, putative | 346 |
| 43 | 46.m03956 | fructose-bisphosphate aldolase, putative | 326 |
| 44 | 55.m08205 | X-prolyl aminopeptidase, putative | 323 |
| 45 | 583.m00019 | hypothetical protein | 322 |
| 46 | 33.m01348 | aggrecan core protein-related | 319 |
| 47 | 57.m00001 | hexokinase | 309 |
| 48 | 38.m01889 | vacuolar ATP synthase subunit B, putative | 305 |
| 49 | 44.m02525 | importin beta-3 subunit, putative | 299 |
| 50 | 20.m03764 | cell wall protein-related | 298 |
| 51 | 49.m03373 | haloacid dehalogenase-like hydrolase domain-containing protein | 295 |
| 52 | 42.m00069 | elongation factor 1-beta, putative | 289 |
| 53 | 50.m03110 | CRAL/TRIO domain-containing protein | 284 |
| 54 | 37.m00743 | glutaminyl-tRNA synthetase, putative | 284 |
| 55 | 583.m05344 | conserved hypothetical protein | 280 |
| 56 | 35.m00026 | ATP-dependent RNA helicase, putative | 279 |
| 57 | 59.m03661 | cell division protein 48, putative | 279 |
| 58 | 55.m00171 | TPR domain-containing protein | 276 |
| 59 | 41.m00006 | eukaryotic translation initiation factor 3 subunit 9, putative | 264 |
| 60 | 38.m01113 | heat shock protein, putative | 261 |
| 61 | 55.m11049 | NAC domain containing protein | 260 |
| 62 | 583.m05554 | 26S proteasome non-ATPase regulatory subunit 2, putative | 259 |
| 63 | 50.m03419 | hypothetical protein | 252 |
| 64 | 52.m00009 | valyl-tRNA synthetase, putative | 252 |
| 65 | 41.m00030 | serine/threonine protein phosphatase, putative | 250 |
| 66 | 50.m03331 | hypothetical protein | 248 |
| 67 | 583.m00615 | thioredoxin reductase, putative | 247 |
| 68 | 641.m00193 | phosphoglycerate kinase, putative | 245 |
| 69 | 583.m00009 | heat shock protein 70, putative | 245 |
| 70 | 65.m01193 | glucose-6-phosphate dehydrogenase, putative | 243 |
| 71 | 583.m05601 | hypothetical protein | 240 |
| 72 | 33.m01287 | peptidase M16 inactive domain-containing protein / insulinase (peptidase family M16) domain-containing protein | 240 |
| 73 | 42.m03350 | aminopeptidase N, putative | 234 |
| 74 | 83.m01195 | NADP-specific glutamate dehydrogenase, putative | 234 |
| 75 | 23.m00001 | ribonucleotide-diphosphate reductase, small subunit, putative | 234 |
| 76 | 583.m05445 | hypothetical protein | 224 |
| 77 | 583.m00606 | hypothetical protein | 218 |
| 78 | 39.m00001 | actin depolymerizing factor | 217 |
| 79 | 50.m00003 | glutathione reductase, putative | 216 |
| 80 | 583.m05329 | long-chain-fatty-acid--CoA ligase, putative | 215 |
| 81 | 55.m05030 | 26S proteasome non-ATPase regulatory subunit 1, putative | 215 |
| 82 | 49.m00027 | D-3-phosphoglycerate dehydrogenase, putative | 212 |
| 83 | 49.m03152 | proteasome subunit alpha type 4, subunit | 212 |
| 84 | 55.m04872 | 60S acidic ribosomal protein P1, putative | 206 |
| 85 | 80.m00002 | phosphoenolpyruvate carboxykinase, putative | 203 |
| 86 | 80.m02344 | HECT-domain (ubiquitin-transferase) containing protein | 197 |
| 87 | 55.m04970 | eukaryotic translation initiation factor 3 subunit 11, putative | 191 |
| 88 | 49.m00020 | ribose 5-phosphate isomerase, putative | 190 |
| 89 | 42.m00106 | conserved hypothetical protein | 190 |
| 90 | 46.m01701 | lysine decarboxylase domain-containing protein | 188 |
| 91 | 80.m02267 | ubiquitin-activating enzyme E1, putative | 186 |
| 92 | 76.m00007 | peptidyl-prolyl isomerase, putative | 178 |
| 93 | 42.m00050 | triosephosphate isomerase, putative | 178 |
| 94 | 50.m00017 | proteasome subunit alpha type 5, putative | 178 |
| 95 | 583.m00642 | hypothetical protein | 177 |
| 96 | 37.m00001 | peroxiredoxin | 174 |
| 97 | 42.m00008 | adenosylhomocysteinase, putative | 172 |
| 98 | 65.m00014 | proteasome subunit alpha type 1, putative | 169 |
| 99 | 41.m00032 | aldehyde dehydrogenase, putative | 164 |
| 100 | 46.m01590 | glycine hydroxymethyltransferase, putative | 162 |
